# The impact of rehabilitation on quality of life after hearing loss: a systematic review

**DOI:** 10.1007/s00405-018-5100-7

**Published:** 2018-08-23

**Authors:** Arjuna Brodie, Bethany Smith, Jaydip Ray

**Affiliations:** University of Sheffield, 30 Regent Street, Sheffield, S1 4DA UK

**Keywords:** Hearing loss, Hearing rehabilitation, Quality of life

## Abstract

**Purpose:**

Hearing loss is a major health problem and is associated with several negative outcomes such as difficulties in communicating and poor quality of life. The aim of this study was to conduct a systematic literature review to evaluate the impact of different types of hearing rehabilitation after hearing loss and their impact on quality of life.

**Methods:**

A systematic literature search was conducted on Pubmed which retrieved 549 articles. Of these, 29 articles regarding cochlear implants, bone anchored hearing devices and traditional amplification hearing aids have been systematically reviewed. The search was limited to articles published from 1960/01/01 to 2017/05/22, included human participants and available in English.

**Results:**

The main finding was that hearing rehabilitation is beneficial in all types of hearing loss and treatment regarding quality of life. However, bone-anchored hearing devices and cochlear implants were shown to produce greater improvements in terms of quality of life than conventional hearing aids.

**Conclusion:**

From these findings, we concluded that hearing rehabilitation does have a positive impact on quality of life after hearing loss.

## Introduction

Hearing loss is a major health problem that often goes un-noticed or untreated. It is associated with an array of problems, such as poor quality of life, negative outcomes with socialisation, independence, interpersonal relationships, socialisation and communications. Figures show that approximately 10 million people in the UK are affected by hearing loss [1].

There are three main types of hearing loss: conductive, sensorineural and mixed. Sensorineural hearing loss (SNHL) is a result of damage to the cochlear or vestibulocochlear nerve [2]. There are different causes of SNHL, such as congenital or acquired, e.g. meningitis and other infections and trauma. Other more common causes are presbycusis and noise-induced hearing loss [3]. Conductive hearing loss (CHL) occurs when conduction is impaired due to a physical or mechanical obstruction to air conduction which inhibits transmission of sound waves from the outer/middle ear to the inner ear [4]. Some causes of CHL include trauma such as a perforated ear drum or infection such as otitis externa and acute or chronic otitis media [5]. There can also be a combination of SNHL and CDHL. This occurs when there is damage in the outer or middle ear along with damage to inner ear, and/or vestibulocochlear nerve and its neural pathways. This is referred to as mixed hearing loss.

In simplistic terms, a hearing aid is an electronic device that is worn behind or in the ear that works by making sounds louder for individuals with hearing loss to hear better. This is done via three basic components: a microphone, an amplifier and a speaker. Sound is received through the microphone, which converts it into electrical signals; these electrical signals are transported to the amplifier which is responsible for increasing the power of the signals which are projected through the speaker [6].

The “bone anchored hearing aid*”* (BAHA) is a type of hearing aid that requires surgical implantation. During surgery, a small vibrator is plugged on to a titanium screw (fixture) that is implanted behind the ear. These two components convert sound into a vibration via the screw, which then stimulates the cochlea through an alternative conduction pathway [7]. The BAHA is generally used as an alternative to traditional hearing aids for conductive or mixed hearing loss. For some individuals a conventional hearing aid can prove to be problematic, therefore, a BAHA can be used [7]. A cochlear implant is another alternative electronic hearing device that is used when a conventional hearing aid cannot be used. The cochlear implant is used to bypass missing or damaged hair cells located in the cochlea that would normally code sound [8].

Hearing loss is a debilitating condition, and as with any disability patients often undergo rehabilitation. American Speech–Language–Hearing Association define hearing rehabilitation as a process where those who suffer with hearing loss are provided treatment and training to help improve their impairment. This is done by helping patients adjust to hearing loss, exploring suitable hearing aids and other devices, and helping them to converse and communicate [9].

Hearing loss can lead to secondary problems such as learning disabilities, social isolation, lack of independence, depression and possible early dementia which all effect quality of life. Valente et al. [10] have well documented that hearing impairments can have negative effects on an individual’s life if left untreated, for example, non-auditory aspects of life are affected, such as decrease in psychological well-being, ability to function in social situations, reduced self-esteem and a reduction in quality of life in general [2, 10, 11].

## Methods

### Aims

This systematic review aims to investigate the different types of hearing rehabilitation and whether the rehabilitation improves quality of life, as well as audiological improvements.

### Search strategy

The literature search was divided into different domains and the following search criteria was implemented on Pubmed: (hearing loss OR presbycusis OR single sided deafness OR conductive hearing loss OR sensorineural hearing loss) AND (quality of life OR impact) AND (rehabilitation OR treatment OR hearing aid OR bone conduction). These terms were to be included in the title or the abstract. Articles were only included if they were in full text, published from 1960/01/01 to 2017/05/22, included human participants and the publication was available in English.

**Fig. 1:**
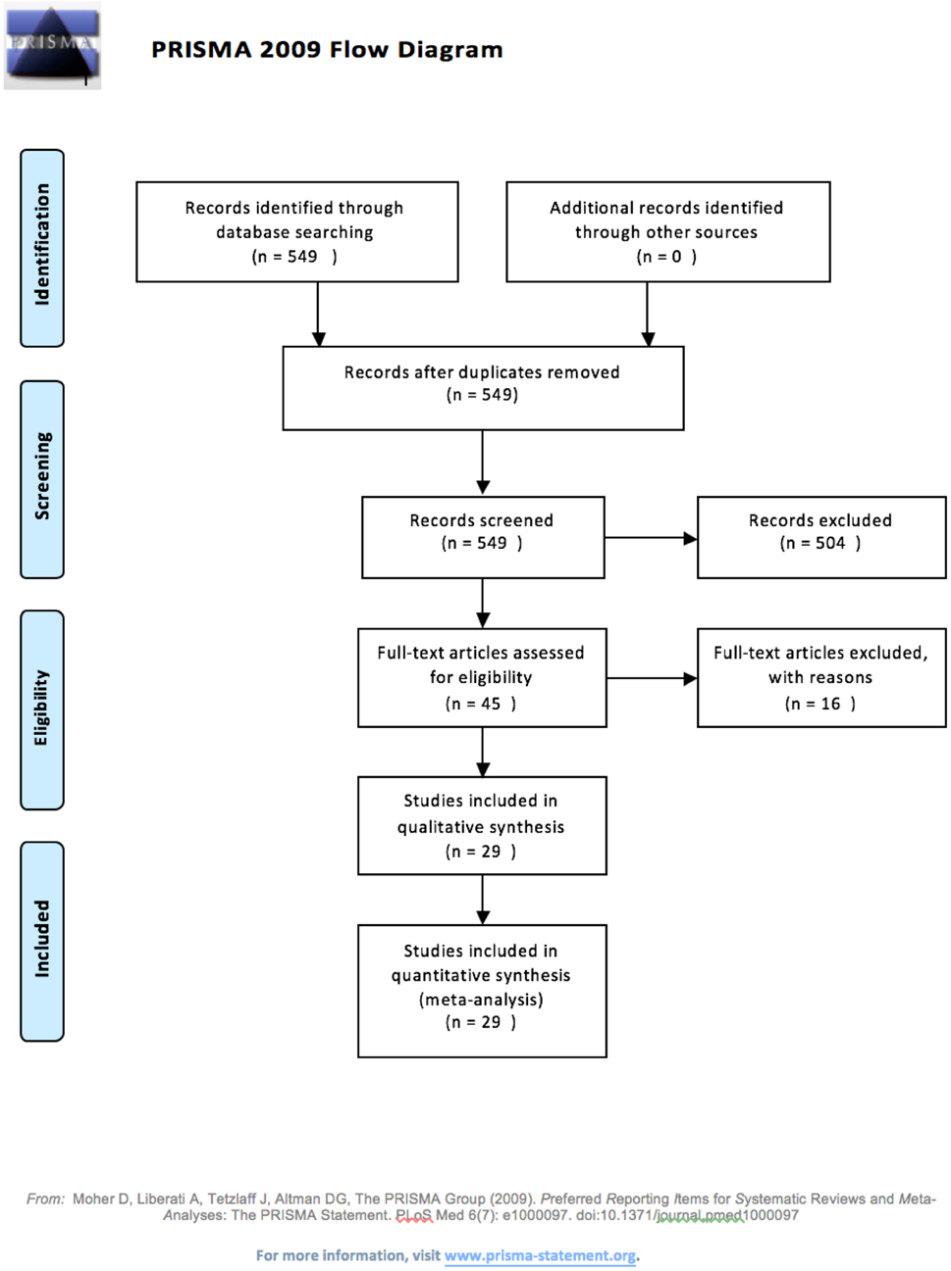
Flow of information through the different phases of a systematic review

The search retrieved 549 articles, which were carefully narrowed down to 45. The majority of these articles were excluded after reading title and abstract as they had no relevance to the research question. Of the 45 articles which had their full-text read, 29 were included in the results. The main reason for exclusion at this stage was due to different outcome measures than quality of life such as hearing improvement solely (see Fig. 1).

## Results

### Cochlear implants

A cochlear implant is an alternative electronic device that is used in profound hearing loss when a conventional amplification hearing aid had little or no benefit or cannot be used. Aimoni et al. [12] conducted a case–control paradigm to assess whether cochlear implants influenced quality of life in 57 participants aged over 65. 42 participants were assigned to the case group and 15 a control group. All participants had been fitted with a cochlear implant to treat profound hearing loss. Audiological data and Quality of Life data via the glasgow benefit inventory (GBI) was collected on four occasions: 1 month before implantation; 1 day pre-implantation; 30 days post-implantation; 12 months post-implantation. Their results showed significant improvements in post implant scores in both their audiological tests and quality of life [12]. Hilly et al. [13] researched into a very similar area in their retrospective chart review of cochlear implants. They recruited 87 participants aged over 60. They investigated the effects of cochlea implants 5 years post implantation which revealed that none of their audiometry scores had declined; they had either stabilised or improved, which in turn had improved participants overall quality of life [13].

Cosetti et al. [14] conducted a longitudinal study in which they researched the impact of cochlear implants on the cognitive functioning of elderly female patients. Though they only recruited seven patients, their results showed that 45% of the participants showed moderate to pronounced improvement, with the memory and verbal being the most successful domains [14]. This is an important piece of research as it highlights that cochlear implants in elderly women improves areas of cognitive functioning.

Necula et al. [15] looked at the quality of life after cochlear implantation in comparison to after traditional hearing aid fittings in children. This was measured by the Nijmegen cochlear implant HRQoL questionnaire, which was sent to parents. 84 of the participants were using cochlear implants and 50 participants were using hearing aids. Their results revealed that cochlear implants showed the greatest improvements in speech production and audiometry performance in comparison to a standard hearing aid [15].

Francis et al. [16] proposed an alternative hypothesis on what makes cochlear implantation successful; they suggested that clinical and psychological factors can determine how well an individual adapts to their cochlear implant. Their results showed that poor education, residing in assisted living facilities and poor general health had a negative impact on speech perception [16]. Therefore, health and psychosocial factors have an impact on successful cochlear implants.

Cochlear implantation is a surgical procedure, and with any surgery comes concern and worry about complications. Estomba et al. [17] conducted a retrospective analysis of patients over the age of 18 who all underwent cochlear implant surgery to review if there were any complications post-surgery. Of the 57 participants, 24.6% reported to have experienced minor complications and 17.5% reported major complications. The most frequently reported minor complication was a vestibular disorder, and the most frequent major complication was device failure. The researchers were able to conclude from there results that the cochlear implant remains a safe surgical technique for rehabilitating hearing loss [17].

### Bone anchored hearing aids

The BAHA plays a role in an array of psychological and personal benefits, such as quality of life, comfort, practicality, cosmetic appearances, anxiety and depression improvements, more social interactions and less social isolation. Arunachalam et al. [18] used the glasgow benefit inventory (GBI) to conduct a retrospective questionnaire study to measure quality of life after BAHA implants. 60 participants were recruited, and their results revealed the BAHA had greatly improved quality of life. When looking into general benefits, the mean score was + 34, the mean score for social benefit was + 21, and the mean scores for physical benefits was + 10 [18].

Further research by Hol et al. [19] was conducted using the health survey (SF-36), the hearing handicap and disability inventory (HHDI) and the EuroQoL-50 (EQ-50) questionnaire. In total, 56 adult participants were recruited, all of whom completed the questionnaires before surgery and 6 months after their BAHA was fitted. The results showed significant improvements in certain areas of participants lives after surgery. For example, the HHDI highlighted significant improvements regarding their handicap and disability, which increased their quality of life. Furthermore, significant improvements in the mental health domain from the SF-36 questionnaire were also found [19].

In addition, De Wolf et al. [20] conducted a retrospective questionnaire study to investigate if the BAHA had the same success for quality of life when administrated to children with unilateral or bilateral hearing impairments. The 31 children were split into three groups: 10 had bilateral conductive hearing loss with normal cognition (BHL-NC); 6 had bilateral conductive hearing loss with mental disability (BHL-MD); 15 had unilateral hearing loss (UHL). Results from the abbreviated profile of hearing aid benefit (APHAB), revealed 70% of the BHL-NC found it to be beneficial. Results did show the younger the patient was when fitted with the BAHA, the more they found it to be beneficial. Glasgow children’s benefit inventory (GCBI) also showed an overall benefit for all three groups, however, greater benefits were reported in the BHL-NC and UHL groups [20]. Similarly, Doshi et al. [21] conducted a retrospective case review to investigate the quality of life outcomes after BAHA surgery in children with single sided sensorineural deafness. The GCBI questionnaire was used and the results showed all, but one of the eight children reported a positive GCBI score [21]. Although the sample size is very small, and therefore, not generalizable, these findings support De Wolf et al., research that the BAHA is an effective form of hearing rehabilitation for children, especially when they have a diagnosis of single-sided deafness.

Gillett et al. [22] received responses to a retrospective postal questionnaire from 41 patients aged between 6 and 88 years old in a district general hospital. The results revealed that quality of life, as measured by the GBI, significantly improved post implant. Furthermore, there were no major complications reported, only minor, with 33% reporting minor temporary skin infections and 17% suffered thickening of the skin around the implant [22]. This research further supports that the BAHA remains a safe, effective and reliable treatment with minimal risks, even when it is run in a smaller district general hospital.

It is important to understand the long-term effects of the BAHA device on quality of life. Newman et al. [23] performed a prospective clinical study on eight participants to analyse the long, medium and short-term benefits and satisfaction for patients fitted with the BAHA to treat profound unilateral SNHL. The results were based on a 95% confidence interval for unaided testing which showed significant improvements in speech perception. They also revealed that participants reported to be overall satisfied with their BAHA in the long-term and would still elect to undergo this procedure a second time. From these results, they were able to conclude that the BAHA is successful in reducing psychosocial consequences of profound unilateral SNHL in the long-term [23].

Carr et al. [24] highlight how it has previously been suggested that the bone conduction hearing aid in an elderly population can lead to more complications, and therefore, reduce the quality of life benefits that have been discussed in detail. To test this, they conducted a retrospective case note review with a telephone and postal questionnaire. 51 participants aged over 60 took part and received implantation due to single-sided deafness, mixed or conductive hearing loss. The outcome measures were rates of complication and quality of life measured by the GBI. The results revealed that the global GBI scores were 82% and the satisfaction scores were 70%. These results strongly disprove the hypothesis that the BAHA is not as effective in elderly patients, the results demonstrate the opposite—bone conduction hearing aids are in fact a reliable and ideal method of rehabilitating a variety of different hearing impairments in the elderly [24].

### Hearing aids

A conventional amplification hearing aid is one of the most frequent devices used to rehabilitate hearing loss. Stewart et al. [25] conducted a prospective longitudinal outcome-based study to measure hearing specific status and quality of life in conductive hearing loss before and after hearing aid and surgery treatments. Results showed that significant improvements in the participants hearing threshold was found in all participants, regardless of the type of treatment. Those treated with a hearing aid demonstrated lower baseline quality of life and hearing status in comparison to those treated with surgery—they also showed decline in quality of life and partial improvement in hearing-specific functional status after treatment [25]. These results are important in demonstrating that hearing-specific functional status in conductive hearing loss can be improved with treatment, although surgical treatment has shown to be more promising.

However, Murlow et al. [26] highlight how hearing loss in the elderly effects quality of life due to lack of communication. They recruited 188 participants with hearing loss. The participants were randomly assigned to receive a hearing aid or join a waiting list. For both groups a generic quality of life and comprehensive battery of disease-specific measures were taken at baseline, 6 weeks and 4 months. Their results revealed that those assigned to receive a hearing aid significantly improved in all areas: social and emotional function (*p* ≤ 0.0001); communication function (*p* ≤ 0.0001); cognitive function (*p* = 0.008); depression (*p* = 0.03) [26]. These results support the hypothesis that hearing loss has negative effects on quality of life that are reversible with hearing aid rehabilitation.

Similarly, Lotfi et al. [27] conducted a study to investigate the quality of life in elderly people who are hard of hearing after they have been fitted with a hearing aid. The 207 participants completed the Hearing Handicap Inventory for the Elderly (HHIE) questionnaire to determine the severity of their hearing loss and their specific communication problems and quality of life. The results from the questionnaire reveal there was a significant difference when measuring quality of life before and 3 months after receiving a hearing aid (*p* ≤ 0.000) [27]. These results are important in highlighting that hearing aids are extremely beneficial in treating presbycusis in the elderly population regarding their communication, and therefore, their quality of life.

A more recent study by Niemensivu et al. [28] conducted a study where they evaluated health-related quality of life in those with a hearing impairment before and after receiving a hearing aid. They recruited 949 adults with hearing impairments. Data were collected with the use of the 15D instrument before and 6 months after hearing aid rehabilitation. The results revealed that those with hearing loss had significantly poorer health-related quality of life on most dimensions both before and after rehabilitation in comparison to the control group, which consisted of the general population. Furthermore, rehabilitation with a hearing aid significantly improved mean scores on the dimensions of hearing, however, the improvement of the overall score was marginal [28]. These results are supportive of the theory that using hearing aids can improve subjective hearing and marginally improve health-related quality of life in adults with hearing impairments. It also suggests that the consequences of hearing loss drastically reduce health-related quality of life when compared to the general population and a hearing aid can only marginally improve this. This implies that, while rehabilitation with a traditional amplification hearing aid does have the ability to improve health-related quality of life in those with hearing impairments, rehabilitation cannot improve it to a standard that places them with the general population.

Chen et al. [29] investigated the impact of sudden SNHL on mental health in adults. They recruited 147 participants who were all admitted with sudden SNHL. They measured the degree of mental distress after a follow-up of around 1 year. They also measured the association between mental distress, tinnitus and hearing recovery. Their results showed that those who recovered from their hearing loss reported significantly less symptoms of depression. Furthermore, those who had tinnitus because of sudden SNHL reported more disruptive personal relationships, disruptive activities, more physical symptoms and more depressive feelings and thoughts [29]. These results highlight the importance of rehabilitation and treatment of sudden SNHL. These results are replicated by Carlsson et al. [30] who investigated the same issue, but with a bigger sample size of 369. They also found that tinnitus was the strongest predictor of negative effects on quality of life. From this study, they were able to conclude that those who suffer with sudden SNHL require extensive hearing rehabilitation from a multidisciplinary angle, such as psychological, medical and social approaches [30]. Heine and Browning [31] add that improved rehabilitation programs that provide carers and clients with strategies to overcome communication breakdown is essential in dealing with the impact of hearing loss. Furthermore, better staff education and a multidisciplinary approach would help to improve the process too [31].

### Other possible causes for quality of life changes

Schneider et al. [32] investigated the impact of hearing loss in the community and the patients informal support. The study included 2956 patients with hearing loss. The cross-sectional analysis showed that those with mild–severe hearing loss demonstrated an 80% increased reliance on informal or formal support, which suggests that hearing impairments increase the need for support. Furthermore, those with hearing loss who did not use a hearing aid were twice as likely to rely on their community support services than those without hearing loss. These findings suggest that hearing loss has a negative impact on independence levels and thus quality of life in older people as they heavily rely on family and their community for support [32]. Perhaps earlier detection and diagnosis of hearing loss would allow for more successful treatment and rehabilitation to help retain independence and cognition which is vital in improving quality of life [24, 32]. However, a more positive outlook of these results is that the reliance they have on their community and other support ensures that the individuals with hearing loss remain social and do not become isolated, which can have a positive effect on quality of life.

## Discussion

From the results it is evident that hearing rehabilitation impacts quality of life in an array of different ways. When considering cochlear implants, several different measures have revealed how the device is beneficial in terms of treating hearing loss and improving quality of life. Furthermore, research highlights how the minimalistic surgery has few risks and several benefits that are long lasting [17].

Regarding the research investigating the BAHA, the results are similar to that of the cochlear implant. Quality of life is consistently improved throughout a large range of different research methodologies which highlights how reliable and consistent the BAHA is. It has been shown to improve and prevent social isolation and learning difficulties in children later in life [20]. Furthermore, a lot of patients report high levels of satisfaction and the positive effects have been found to be long-lasting in longitudinal studies [23]. The BAHA device is essential in preventing isolation from inability to communicate and preserving cognition in a growing elderly population [24].

While the research on conventional hearing aids has not been as profound as the other research in terms of quality of life improvement, it does play an important role preserving hearing abilities [27]. As well as rehabilitation with hearing devices, external and alternative sources have been shown to be essential in maintaining psychological well-being and ensuring compatibility with the device. For example, increasing knowledge in order for hearing loss symptoms to be detected earlier [20], providing psychological and social support to help adapt to a new device, promoting positive behaviours [16] and attitudes and making hearing loss a more urgent disability are all important factors that the general population, medical professionals and personal carers need to work towards to improve hearing rehabilitation [30, 31].

### Conclusion

In summary, there is a large body of research into the impact of hearing loss rehabilitation and quality of life. The majority of it states that the results of hearing rehabilitation are largely positive. A hearing device is no longer just available to improve auditory health, instead increased knowledge and awareness has opened our eyes in our understanding to hearing loss, in the sense that there are many different areas of our lives that can be affected from hearing loss. Therefore, successful hearing rehabilitation can have a profound impact on quality of life.

## References

[1] Hearing Link (2011) Facts about deafness and hearing loss. https://www.hearinglink.org/your-hearing/about-deafness-hearing-loss/facts-about-deafness-hearing-loss/. Accessed 17 July 2017

[2] Chisolm TH, Johnson CE, Danhauer JL, Portz LJP, Abrams HB, Lesner S (2007). A systematic review of health-related quality of life and hearing aids: final report of the American Academy of Audiology Task Force on the health-related quality of life benefits of amplification in adults. J Am Acad Audiol.

[3] Bansal M (2013). Disease of the Ear Nose and Throat.

[4] Ali W, Suebwongpat A, Weston A (2008). The effectiveness of digital hearing aids and assistive listening devices for adults with hearing loss: a systematic review of the literature. HSAC Rep.

[5] Hussain MSSM (2008) Conductive hearing loss, Nottingham, pp 3–7. Retrieved from https://assets.publishing.service.gov.uk/government/uploads/system/uploads/attachment_data/file/384492/conductive_hearing_loss.pdf. Accessed 6 Aug 2017

[6] NIDCD (2017) Hearing and balance: hearing aids. https://www.nidcd.nih.gov/sites/default/files/Documents/health/hearing/nidcd-hearing-aids.pdf. Accessed 17 July 2017

[7] Forton GEJ, Van de Heyning PH (2007). Bone anchored hearing aids (BAHA). B-ENT.

[8] Cunningham RF (2017) A resource guide for early hearing detection and intervention: cochlear Implants. http://libguides.dixie.edu/c.php?g=57887&p=371717. Accessed 17 July 2017

[9] American Speech-Language-Hearing Association (2015) Audiological (hearing) rehabilitation. http://www.asha.org/public/hearing/Audiologic-Rehabilitation/. Accessed 17 July 2017

[10] Valente M, Abrams H, Benson D, Chisolm T, Citron D, Hampton D (2006). Audiologic management of adult hearing impairment. Audiol Today.

[11] Taylor B (2006). How quality of service affects patient satisfaction with hearing aids. Hear J.

[12] Aimoni C, Ciorba A, Hatzopoulos S, Ramacciotti G, Mazzoli M, Bianchini C, Rosignoli M, Skarżyński H, Skarżyński PH (2016). Cochlear implants in subjects over age 65: quality of life and audiological outcomes. Med Sci Monit.

[13] Hilly O, Hwang E, Smith L, Shipp D, Nedzelski JM, Chen JM, Lin VW (2016). Cochlear implantation in elderly patients: stability of outcome over time. J Laryngol Otol.

[14] Cosetti MK, Pinkston JB, Flores JM, Friedmann DR, Jones CB, Roland JT, Waltzman SB (2016). Neurocognitive testing and cochlear implantation: insights into performance in older adults. Clin Interv Aging.

[15] Necula V, Cosgarea M, Necula SE (2012). Health-related quality of life in cochlear implanted patients in Romania. Int J Pediatr Otorhinolaryngol.

[16] Francis HW, Yeagle JA, Thompson CB (2015). Clinical and psychosocial risk factors of hearing outcome in older adults with cochlear implants. Laryngoscope.

[17] Estomba CCM, Schmitz TR, Reinoso FAB, Collado LD, Garcia ME, Lorenzo AIL (2016). Complications after cochlear implantation in adult patients. 10-year retrospective analysis of a tertiary academic centre. Auris Nasus Larynx.

[18] Arunachalam PS, Kilby D, Meikle D, Davison T, Johnson IJ (2001). Bone-anchored hearing aid quality of life assessed by Glasgow Benefit Inventory. Laryngoscope.

[19] Hol MK, Spath MA, Krabbe PF, Van Der Pouw CT, Snik AF, Cremers CW, Mylanus EA (2004). The bone-anchored hearing aid: quality-of-life assessment. Arch Otolaryngol Head Neck Surg.

[20] De Wolf MJ, Hol MK, Mylanus EA, Snik AF, Cremers CW (2011). Benefit and quality of life after bone-anchored hearing aid fitting in children with unilateral or bilateral hearing impairment. Arch Otolaryngol Head Neck Surg.

[21] Doshi J, Banga R, Child A, Lawrence R, Reid A, Proops D, McDermott AL (2013). Quality-of-life outcomes after bone-anchored hearing device surgery in children with single-sided sensorineural deafness. Otol Neurotol.

[22] Gillett D, Fairley JW, Chandrashaker TS, Bean A, Gonzalez J (2006). Bone-anchored hearing aids: results of the first eight years of a programme in a district general hospital, assessed by the Glasgow benefit inventory. J Laryngol Otol.

[23] Newman CW, Sandridge SA, Wodzisz LM (2008). Longitudinal benefit from and satisfaction with the Baha system for patients with acquired unilateral sensorineural hearing loss. Otol Neurotol.

[24] Carr SD, Moraleda J, Baldwin A, Ray J (2016). Bone-conduction hearing aids in an elderly population: complications and quality of life assessment. Eur Arch Otorhinolaryngol.

[25] Stewart MG, Coker NJ, Jenkins HA, Manolidis S, Bautista MH (2000). Outcomes and quality of life in conductive hearing loss. Otolaryngol Head Neck Surg.

[26] Mulrow CD, Aguilar C, Endicott JE, Tuley MR, Velez R, Charlip WS, Rhodes MC, Hill JA, DeNino LA (1990). Quality-of-life changes and hearing impairment. A randomized trial. Ann Intern Med.

[27] Lotfi Y, Mehrkian S, Moossavi A, Faghih-Zadeh S (2009). Quality of life improvement in hearing-impaired elderly people after wearing a hearing aid. Arch Iran Med.

[28] Niemensivu R, Manchaiah V, Roine RP, Kentala E, Sintonen H (2015). Health-related quality of life in adults with hearing impairment before and after hearing-aid rehabilitation in Finland. Int J Audiol.

[29] Chen J, Liang J, Ou J, Cai W (2013). Mental health in adults with sudden sensorineural hearing loss: an assessment of depressive symptoms and its correlates. J Psychosom Res.

[30] Carlsson PI, Hall M, Lind KJ, Danermark B (2011). Quality of life, psychosocial consequences, and audiological rehabilitation after sudden sensorineural hearing loss. Int J Audiol.

[31] Heine C, Browning CJ (2002). Communication and psychosocial consequences of sensory loss in older adults: overview and rehabilitation directions. Disabil Rehabil.

[32] Schneider J, Gopinath B, Karpa MJ, McMahon CM, Rochtchina E, Leeder SR, Mitchell P (2010). Hearing loss impacts on the use of community and informal supports. Age Ageing.

